# Stress–Strain and Dimension Evolution of Wind Turbine Bearing Ring with Non-Standard Section During Hot Bulging Process

**DOI:** 10.3390/ma19050938

**Published:** 2026-02-28

**Authors:** Ruijie Gu, Yutong Fu, Ziyang Shang, Zhuangya Zhang, Shan Lan, Tongxun Wang, Qiang Wang, Liaoyuan Chen

**Affiliations:** 1 School of Mechatronics Engineering, Henan University of Science and Technology, Luoyang 471003, China; 2 State Key Laboratory of Intelligent Mining Heavy Equipment, Luoyang 471003, China; 3 LYC Bearing Group Corporation, Luoyang 471000, China; 4 School of Intelligent Manufacturing and Electrical Engineering, Nanyang Normal University, Nanyang 473001, China

**Keywords:** bear rings, non-standard section, stress–strain, dimension evolution, stepwise rotation, bulging process

## Abstract

As wind turbines trend toward larger sizes, higher rotational speeds, and extended service lives, higher demands are emerging for the dimensional accuracy, mechanical properties, and service reliability of the main shaft bearings. The hot bulging process is a critical process in bearing ring manufacturing. The stress–strain and dimensional evolution during the hot bulging process are crucial for the fatigue life and dimensional accuracy of rings with non-circular cross-sections. Therefore, based on the residual stress field from rolling as an initial condition, this paper established a coupled finite element model for the entire rolling-to-bulging process of GCr15SiMn bearing steel rings and verified the accuracy of the model. A stepwise rotation hot bulging process was innovatively designed. The stresses, strains, and deformation rates of the rings were thoroughly evaluated at different steps of the bulging process. Additionally, the effect of the bulge amount on the stress–strain uniformity and dimensional accuracy of the fabricated rings was also evaluated. Results indicate that based on the stepwise rotation hot bulging process conducted at 870–930 °C, when the first-step bulging amount is 1.50 mm, the secondary and third-step amounts are both 0.50 mm, and the bulging speed is 1.00 mm/s, while the roundness error of ring #3 stabilizes within 0.28–0.35 mm. The standard deviation of the axial equivalent strain was decreased by 92%, and the stress peak was also decreased by 39%. Above all, the stepwise rotation hot bulging process is an effective approach to improve the distribution uniformity of the stress–strain and the dimensional consistency of the bearing rings. This paper provides theoretical foundations and process guidance for the precision forming of large wind turbine bearing rings with non-standard sections.

## 1. Introduction

As wind turbines evolve toward larger scales, the loads borne by their transmission systems increase substantially in both magnitude and complexity [1,2]. Therefore, the main bearing supports radial and axial loads as well as complex moments while also efficiently transmitting torque [3,4,5,6,7]. The manufacturing of large wind turbine main shaft bearing rings with high dimensional accuracy, superior mechanical properties, and exceptional reliability therefore constitutes a pivotal challenge for advancing technological progress and cost control in the wind power industry. The rolling process, as an efficient continuous local forming method, is applied in the near-net-shape manufacturing of large rings. Numerous studies indicate that the residual stresses released and accumulated during the forming of bearing rings [8] are closely related to high-precision dimensional requirements and long-term stable operational performance. However, for large wind turbine main shaft bearing rings with non-standard sectional geometries and stringent dimensional accuracy requirements, issues such as non-uniform residual stress and insufficient dimensional precision inevitably arise during the manufacturing stage, thereby increasing uncertainties in subsequent machining and assembly processes [9]. Therefore, there is an urgent need to introduce a secondary forming process after rolling that can synergistically achieve both stress regulation and dimensional finishing. The bulging process, which induces local plastic deformation in bearing rings by actively applying external pressure, effectively adjusts the distribution of residual stresses and reduces their peak values. Simultaneously, it enables a slight plastic correction of the ring’s internal and external contours, significantly enhancing dimensional accuracy and geometric stability. As a result, it has become an important technical method for regulating residual stresses post-rolling [10]. The investigation of the combined “rolling–bulging” process is of significant engineering importance for achieving the high-performance and high-precision manufacturing of large wind turbine main shaft bearing rings.

To gain a deeper understanding of the relationship between stress–strain behavior and dimensional evolution during the ring bulging process, numerous scholars have conducted extensive research. The bulging process serves as a critical post-forming technique employed to achieve the stress homogenization and dimensional calibration of bearing rings. Mei et al. [11] analyzed the limitations of traditional rolling processes to address issues such as significant residual stress and unstable dimensional accuracy in wind power bearing rings after rolling, and they innovatively proposed a matched “rolling–bulging” process. Results indicated that the bulging process is capable of effectively achieving the required objectives of stress relief and dimensional finishing for post-rolling rings. This approach is particularly well suited for enhancing the dimensional accuracy and service performance of large rings with non-standard cross-sections. Lan et al. [12] established a finite element model for a multi-step cold bulging process applied to large aluminum alloy rings with non-standard cross-sections. The issue of uneven strain distribution caused by the die contact friction was found to be significantly mitigated by employing a combined strategy of stepwise bulging and circumferential rotation. The cyclic operation of “bulging–rotation” effectively promotes the homogenization of circumferential and axial strains in the ring, providing a key process route for achieving high roundness and dimensional stability. Zhu et al. [13] conducted finite element simulations and experimental validation on the vacuum hot bulging process for Hastelloy C-276 thin-walled cylindrical components. By establishing a high-temperature creep constitutive model for the material and integrating it into a finite element solver, the study successfully predicted the final dimensions of the workpiece under the influence of thermal stress relaxation. Yang et al. [14] proposed a cross-sectional geometric model based on an outer circular and inner elliptical shape to address the forming limit issues of non-uniform thickness tubes during hydroforming. By integrating finite element simulation with three instability criteria—strain variation, strain rate variation, and strain path variation—they established forming limit diagrams for SUS304 stainless steel tubes with non-uniform thickness. Cai et al. [15] investigated the influence of flow stress calculation on the deformation behavior of aluminum alloy sheets based on warm bulge tests. The sphericity of the bulged specimens’ profiles was evaluated using the least squares circle fitting method. Theoretical models with various curvature radii and thicknesses were comparatively analyzed, and a combined model suitable for a specified range of height-to-diameter ratios was ultimately proposed for calculating flow stress curves. Cui et al. [16] investigated the bulging behavior of thick-walled 6063 aluminum alloy tubes under dual-side pressure, analyzing the influence of external pressure on the limiting expansion ratio, bulged zone profile, and thickness distribution through experiments and simulations. The results indicate that while external pressure has a negligible effect on the deformation behavior of the uniform bulging zone, it significantly increases the thickness strain in the fracture zone and improves the fracture limit. Yang et al. [17] proposed introducing a cold bulging process between quenching and aging treatments for 2219 aluminum alloy rings to reduce the quenching residual stress. The influence of the bulging ratio (1–5%) and the number of bulging cycles (two to three times) on the magnitude and distribution uniformity of residual stress was investigated. The results indicate that incorporating the cold bulging process can reduce residual stress by over 85%. However, an excessive bulging ratio (e.g., 5%) can lead to stress deterioration due to die clearance. While the studies have made significant progress in areas such as process parameters for ring bulging, dimensional prediction, and residual stress control, several limitations remain. On one hand, existing research predominantly focuses on the bulging behavior of uniform cross-sections. For wind turbine bearing rings with non-standard cross-sections, the stress concentrations and non-uniform dimensional evolution laws induced by abrupt geometric changes during the bulging process remain unclear. On the other hand, most studies overlook the decisive influence of the residual stress field generated by the preceding rolling process on the stress–strain evolution during bulging. They fail to address the inheritance and evolution of the residual stress field throughout the entire rolling-to-bulging process and its mechanism of action on the final dimensional accuracy. Finally, existing research often treats stress–strain analysis and dimensional accuracy evaluation as relatively separate aspects, lacking investigation into how the dynamic evolution of stress and strain influences macroscopic dimensional precision.

Above all, this paper proposes a continuous simulation and experimental methodology for the rolling-to-bulging process. Firstly, a finite element model for rolling rings with non-standard cross-sections was established to obtain the post-rolling geometric dimensions and residual stress characteristics, which serve as the initial conditions for the bulging process. The simulation results for rolling are then experimentally validated. Second, the stepwise rotation hot bulging process was designed. Furthermore, the regulatory mechanism of the bulging amount on ring dimensional accuracy is systematically investigated. Finally, based on the simulated results, the inherent causal relationships among strain, stress, and ring dimensions are clarified by extracting the stress–strain data at key points and the evolution of cross-sectional dimensions. This provides a direct theoretical basis and optimization direction for formulating the bulging process for rings with non-standard cross-sections.

## 2. Modeling the Hot Bulging Process of Bearing Ring

### 2.1. Theoretical Methods of Modeling

The hot bulging process of bearing rings with non-standard cross-sections primarily achieves precise control over the key geometric dimensions of the ring through near-net-shape manufacturing methods. Therefore, the metallic material undergoes stages of elastic deformation, elastoplastic deformation, and plastic deformation [18,19,20,21], involving complex multi-physics fields characterized by thermo-mechanical coupling and contact-induced nonlinearities in the interaction zones. During the hot bulging process, the deformation can be considered quasi-static due to the relatively low bulging speed [22]. Consequently, the internal stress state during ring deformation satisfies static equilibrium, which can be expressed as

(1) $$\frac{\partial \sigma_{ij}}{\partial x_{j}}+b_{i}=0\;\left(i,j=1,2,3\right)$$

where *σ_ij_* represents the components of the Cauchy stress tensor, describing the internal stress state of the object; and *b_i_* denotes the components of the body force per unit volume.

This paper employs logarithmic strain to address the large deformation of metallic materials during the hot bulging process. The true elongation and compression of the material are reflected by defining the deformation gradient. Under the assumptions of incremental form and small strain, the relationship between the strain tensor and the displacement field can be expressed as [23]

(2) $$\varepsilon_{ij}=\frac{1}{2}\left(\frac{\partial u_{i}}{\partial x_{j}}+\frac{\partial u_{j}}{\partial x_{i}}\right)$$

where *ε_ij_* represents the components of the strain tensor, and *u_ij_* denotes the components of the displacement vector.

Prior to yielding during the hot bulging process, the relationship between stress and strain for the bearing ring can be considered linear and is expressed as

(3) $$\sigma_{ij}=E\left(\frac{\epsilon_{ii}\delta_{ij}}{1-\nu }+\frac{\epsilon_{ij}}{1+\nu }\right)$$

where *E* is Young’s modulus, *υ* is Poisson’s ratio, and *δ_ij_* is the Kronecker delta.

When the stress on the bearing ring reaches the yield strength, irreversible plastic flow begins to occur. This paper adopts the von Mises yield criterion, which is widely used in metal plastic forming and can be expressed as [24]

(4) $$\begin{array}{c} \sigma_{eq}=\sqrt{\frac{1}{2}\left[{\left(\sigma_{11}-\sigma_{22}\right)}^{2}+{\left(\sigma_{22}-\sigma_{33}\right)}^{2}+{\left(\sigma_{33}-\sigma_{11}\right)}^{2}+6\left(\tau_{12}^{2}+\tau_{23}^{2}+\tau_{31}^{2}\right)\right]} \end{array}$$

where *σ_eq_* represents the equivalent stress; *σ*_11_, *σ*_22_ and *σ*_33_ are the normal stress components; and *τ*_12_, *τ*_23_, and *τ*_21_ are the shear stress components.

The direction of the plastic strain increment is determined by the flow rule. This paper adopts the associated flow rule:

(5) $$d\varepsilon_{ij}^{p}=d\lambda \frac{\partial \bar{\sigma }}{\partial \sigma_{ij}}$$

where *dλ* is a non-negative plastic multiplier.

In this paper, the ring bulging process is conducted at elevated temperatures. Consequently, heat conduction occurs between the outer ring and the internal bulging block, while heat convection and radiation take place with the surrounding air. This process is described by [25]

(6) $$\rho c_{p}\frac{\partial T}{\partial t}=\nabla \cdot \left(k\nabla T\right)+Q_{\mathrm{plastic}}+Q_{\mathrm{friction}}$$

among them, the heat generated by plastic deformation and friction are respectively expressed as [26]

(7) $$Q_{\mathrm{plastic}}=\eta \sigma :\varepsilon^{p}$$

(8) $$Q_{\mathrm{friction}}=\mu p_{n}v_{rel}$$

where *T* represents the temperature (°C), ρ represents the material density, *C_p_* represents the specific heat capacity (units), *t* represents the bulging time, *k* is a proportionality coefficient, *Q_plastic_* represents the heat generated by plastic work, *Q_friction_* represents the heat generated by friction, *η* represents the thermal conversion coefficient of plastic work, *σ* represents the flow stress, *ε^p^* represents the plastic strain rate, *μ* represents the friction coefficient, *P_n_* represents the normal pressure on the contact surface, and *V_rel_* represents the relative sliding velocity.

During the hot bulging process, the contact between the ring and the dies constitutes the direct boundary condition determining the final part geometry and dimensional accuracy. To ensure the outer contour of the ring conforms to the die profile for achieving the target cross-sectional shape, the hard contact model is employed to simulate the normal contact behavior between surfaces, which can be expressed as

(9) $$\left\{\begin{array}{c} p=0,g>0\\ p\geq 0,g=0 \end{array}\right..$$

where *p* is the contact pressure and *g* is the contact gap.

The Coulomb friction model is used to describe the interfacial resistance during tangential frictional motion. The contact shear stress has an upper limit determined by the normal pressure of p and the friction coefficient of μ.

(10) $$\tau \leq \tau_{crit}=\mu \cdot p$$

When *τ* < *τ_crit_*, the state is sticking. When *τ* = *τ_crit_*, relative sliding occurs. Therefore, the friction coefficient μ is a critical process parameter. An excessively high friction coefficient can impede metal flow, while insufficient friction may lead to unintended slippage of the ring, resulting in a loss of dimensional control.

The explicit dynamic method advances the computation through numerous small incremental steps, eliminating the need for iterative solutions of the global stiffness matrix. This approach is highly stable and efficient for handling complex contact problems, enabling the accurate acquisition of stress, plastic strain, and temperature states prior to unloading. Therefore, the finite element model for ring hot bulging adopts an explicit dynamic solver whose core involves explicit time integration of the nodal equations of motion using the central difference method [27]:

(11) $$M\ddot{u}\left(t_{n}\right)+C\dot{u}\left(t_{n}\right)+F^{\mathrm{int}}\left(t_{n}\right)=F^{\mathrm{ext}}\left(t_{n}\right)$$

where *M* is the mass matrix, *C* is the damping matrix, and *F_int_* and *F_ext_* are the internal force vector and external force vector, respectively.

### 2.2. Material Properties

**Figure 1:**
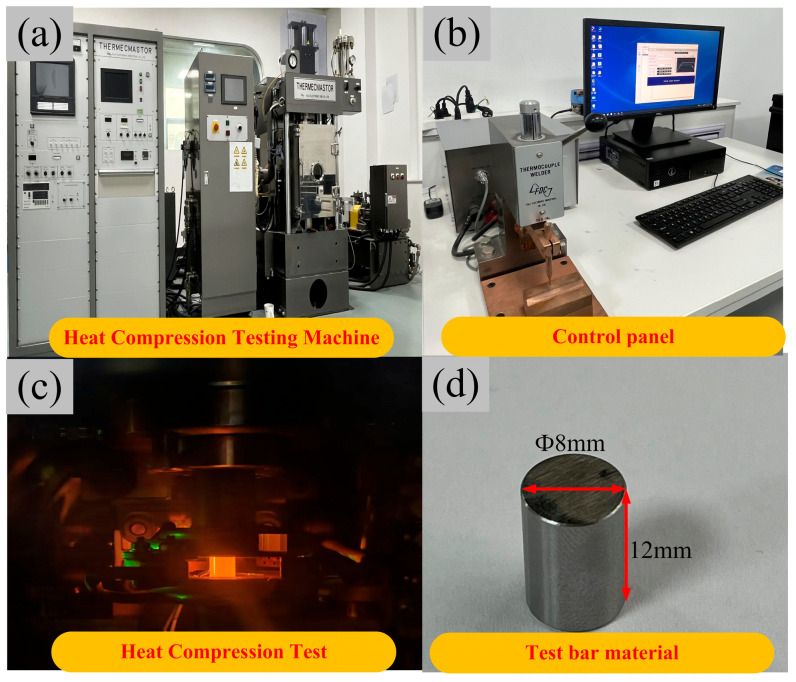
The hot compression testing process for the GCr15SiMn: (**a**) heat compression testing machine, (**b**) control panel, (**c**) heat compression test, (**d**) test bar material.

GCr15SiMn bearing steel subjected to heat treatment can achieve a surface hardness of 60–65 HRC, demonstrating excellent wear resistance and fatigue performance [28]. Consequently, the material selected for this paper is GCr15SiMn, which is commonly used for wind turbine bearing rings. Its chemical composition provided in Table 1. During the hot bulging process of bearing rings, the properties of GCr15SiMn vary with temperature. Therefore, obtaining the true stress–strain data of the material under high temperatures and high strain rates is essential to ensure the accuracy of the constructed simulation model. Therefore, prior to constructing the simulation model, this paper first conducts isothermal hot compression tests on GCr15SiMn bearing steel using a Gleeble-3500 testing machine (Dynamic Systems Inc., Poughkeepsie, NY, USA), as illustrated in Figure 1. This equipment employs direct resistance heating combined with a closed-loop servo-hydraulic control system, enabling high-precision synchronous control of temperature and deformation. Cylindrical specimens (∅8 mm × 12 mm) were machined from the as-received GCr15SiMn bearing steel. K-type thermocouples were spot-welded at the specimen mid-length to monitor and control the temperature.

**Figure 2:**
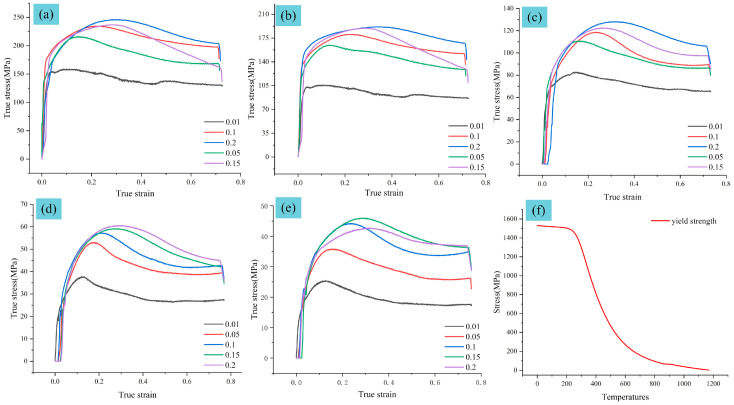
Stress–strain curves of GCr15SiMn bearing steel at different temperatures: (**a**) 710 °C, (**b**) 840 °C, (**c**) 920 °C, (**d**) 1080 °C, (**e**) 1160 °C, (**f**) yield strength.

**Figure 3:**
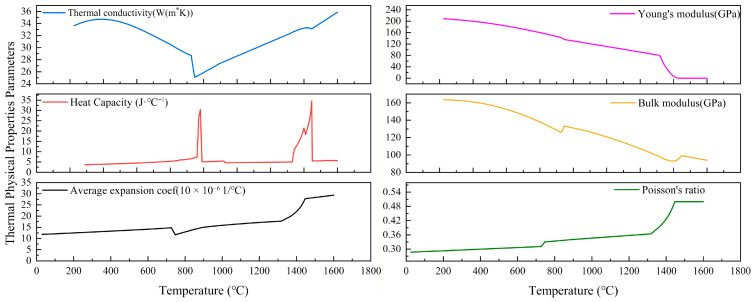
Thermal properties of GCr15SiMn bearing steel.

To comprehensively characterize the material behavior within the hot working window, experiments were designed incorporating three factors: temperature, strain rate, and true strain. The deformation temperatures ranged from 710 °C to 1160 °C, the strain rates ranged from 0.005 s^−1^ to 0.2 s^−1^ (specifically 0.005, 0.01, 0.15, and 0.2 s^−1^), and the maximum true strain was set to 0.7. Before the compression test, all specimens are heated at a rate of 10 °C/s to the target temperature and held for 5 min to ensure temperature uniformity. During the isothermal compression tests, the true strain was directly measured by the high-precision linear variable differential transformer (LVDT) integrated within the Gleeble-3500 system, which monitors the axial displacement of the specimen’s gauge section in real time. This method, combined with the synchronous acquisition of load data, allows for the direct derivation of true stress–true strain curves. Unlike methods that estimate strain from cross-head displacement, this direct measurement approach effectively minimizes errors associated with system compliance and machine deflection, thereby ensuring the accuracy of the constitutive data used for subsequent finite element simulations. As shown in Figure 2. the true stress–true strain curves of GCr15SiMn bearing steel are presented for four temperature groups: 710 °C,840 °C, 920 °C, 1080 °C, and 1160 °C. From these curves, the yield strength at each temperature was extracted and is summarized in Figure 2f. Under constant strain rate and strain conditions, the flow stress of the material exhibits a monotonic decreasing trend with increasing deformation temperature. Conversely, under identical strain and temperature conditions, the true flow stress increases significantly with an increase in strain rate. Furthermore, the thermo-physical properties of GCr15SiMn bearing steel shown in Figure 3, including thermal conductivity, specific heat capacity, and thermal expansion coefficient—were calculated using JMatPro software (version 12.0) with the General Steel database. JMatPro employs the CALPHAD (Calculation of Phase Diagrams) method to predict temperature-dependent material properties based on thermodynamic and kinetic models. The calculations were performed over the temperature range of 20–1200 °C under atmospheric pressure, assuming thermodynamic equilibrium. This approach is widely adopted in both academia and industry for estimating the thermophysical properties of advanced alloys.

### 2.3. Boundary Settings of Joint Simulation Model

**Figure 4:**
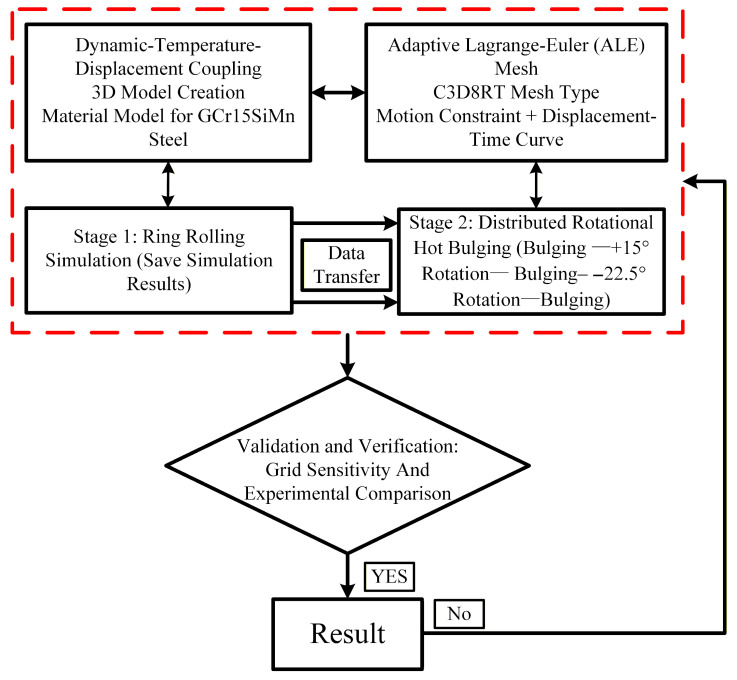
Finite element computation diagram.

This paper employs the commercial finite element software Abaqus/Explicit (Version 2022, Dassault Systèmes, Vélizy-Villacoublay, France) to simulate the integrated rolling–bulging process of the bearing ring; the finite element computation diagram is shown in Figure 4. A dynamic, temperature-displacement coupled explicit integration scheme was adopted to efficiently handle large deformations and complex contact conditions.

To balance computational accuracy and efficiency, an Arbitrary Lagrangian–Eulerian (ALE) adaptive meshing technique was applied to the ring workpiece. This technique automatically remeshes the domain during analysis to maintain element quality. The model was primarily discretized using 8-node thermally coupled brick elements with reduced integration and hourglass control (C3D8RT), which effectively mitigate hourglassing while solving the temperature and displacement fields simultaneously. The model consists of 10,956 elements and 13,363 nodes in total. Local mesh refinement was implemented in the critical contact zones between the ring and the tools (rolls, bulging heads, etc.), with a minimum element size of approximately 3.1 mm × 2.5 mm × 2.65 mm, to ensure the accurate resolution of contact stresses and deformation gradients.

All tooling components (drive roll, conical roll, mandrel, segmented bulging heads) were modeled as analytical rigid bodies. During the rolling stage, the drive roll and conical roll rotate about their own axes, while the mandrel performs a radial feed according to a predefined displacement–time curve to accomplish ring expansion and profiling. During the bulging stage, the segmented bulging heads are synchronously displaced radially following specified displacement–time curves to simulate the bulging operation; the wedge block is assigned an axial displacement to provide the required clamping force. Contact between the ring and the tool surfaces was defined using a “surface-to-surface” contact algorithm with “hard contact” for normal behavior and a Coulomb friction model for tangential behavior. The friction coefficients are listed in Table 2.

The constitutive behavior of the ring material (GCr15SiMn bearing steel) was based on the hot compression experimental data described in Section 2.2. The model defines temperature- and strain-rate-dependent elastoplastic properties, where the flow stress is interpolated from the experimental curves. Additionally, key thermophysical parameters of the material—including thermal conductivity, specific heat capacity, and thermal expansion coefficient (see Figure 4)—were incorporated into the model to couple the heat generation and conduction during deformation.

**Figure 5:**
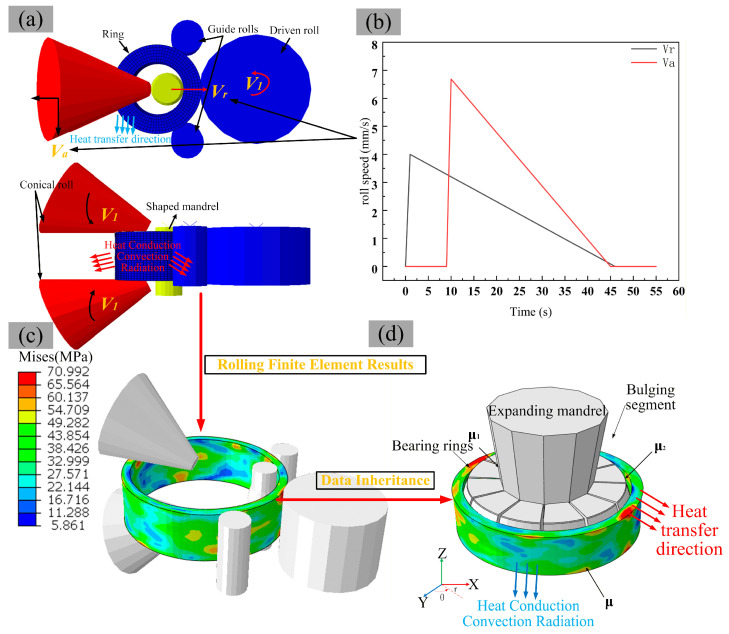
Simulation model of rolling and bulging process of the bearing ring: (**a**) FEM of the ring rolling process, (**b**) schematic diagram of rolling process parameters, (**c**) key parameters of the rolling dies and the post-rolling stress distribution, (**d**) FEM of the bulging process with detailed dimensional parameters of the segmented bulging heads.

Since the hot bulging process of bearing rings with complex profiles is a subsequent step following rolling, the dimensional and stress distributions of the post-rolling rings significantly influence the stress–strain evolution and dimension variation during the bulging process [29]. Therefore, this paper innovatively establishes an integrated simulation model that combines both the rolling and bulging processes, as shown in Figure 5. Based on the rolling equipment for wind turbine bearing rings and the dimensions of the part blank, a three-dimensional model of the rolling process for the bearing outer ring was established (Figure 5a). During the ring rolling process, the driven roller and the conical roller act as driving rolls, rotating around their respective central axes. The rotational speed of the conical roll is dynamically adjusted to match the real-time linear velocity at the end face. The workpiece is rotated via friction between the rolls and the ring, while the shaped mandrel performs a linear radial feed motion. Axial deformation of the ring is cooperatively controlled by the upper and lower conical rolls [29]. Based on the relevant literature [30,31,32,33], reasonable rolling process parameters were derived, and the specific parameters are provided in Figure 5b and Table 2.

As shown in Figure 5d, the bulging model for the bearing ring with a non-standard cross-section employs 12 segmented fan-shaped bulging heads (each with a central angle of 30°). The profiles match the geometric parameters of the raceway in the formed ring. The materials of the bulging head and mandrel are 5CrNiMo and 42CrMo, respectively.

The friction coefficients for the two critical interfaces were set based on their distinct contact conditions. For the mandrel–bulging slider interface (μ_1_), which is a tool-internal sliding contact often maintained with smoother surfaces and possible lubrication in practice, a value of 0.15 was adopted to represent such relatively well-conditioned sliding. For the bulging slider–ring interface, which undergoes dry, high-temperature, high-pressure contact without active lubrication, according to the von Mises yield criterion, and under the common simplifying assumption that *p* ≈ *σy*, the theoretical upper limit for the friction coefficient during plastic forming is between 0.5 and 0.577 [34]. The chosen value μ_2_ = 0.5 approaches this upper range, representing near-sticking conditions in the ring-head contact [30]. To generate a larger radial bulging force with a smaller axial pulling force, the wedge block is designed with a taper angle of 6° (typically within the range of 6–8°).

### 2.4. Mesh Convergence Analysis

To verify the mesh independence and reliability of the finite element simulation results, a systematic mesh convergence analysis was conducted for the model presented in this paper. For the representative ring #3 bulging process model, three mesh schemes were established using global mesh density parameters of 25, 15, and 5 (representing the relative coarseness/fineness of element size) while keeping all other boundary conditions, material parameters, and process settings identical. The complete three-step hot bulging process was simulated for each scheme, and the following key response indicators were extracted and compared: final wall thickness at the L1 path, ring roundness error, average von Mises stress on the cross-section, and strain uniformity (characterized by the standard deviation of equivalent plastic strain).

The analysis results are presented in Table 3. Under different mesh densities, the fluctuations in all output indicators remain at a low level: the wall thickness ranges from approximately 107.35 to 107.95 mm, the roundness error ranges from 0.308 to 0.317 mm, the average stress ranges from 29.98 to 30.28 MPa, and the strain uniformity standard deviation ranges from 0.00342 to 0.00354. Calculations show that the relative variation in all indicators does not exceed 3.5%, which is well below the commonly accepted convergence threshold in engineering simulations (typically 5–10%). This indicates that within the selected range of mesh densities, the simulation results are insensitive to mesh size and exhibit good numerical convergence.

Based on the above analysis, the mesh configuration with a global mesh density parameter of 25 was ultimately selected for all formal simulations in this paper. This scheme ensures result accuracy while maintaining computational efficiency. The data obtained can be regarded as a reliable, mesh-independent solution suitable for subsequent process analysis and conclusion derivation.

## 3. Experiment and Characterization

**Figure 6:**
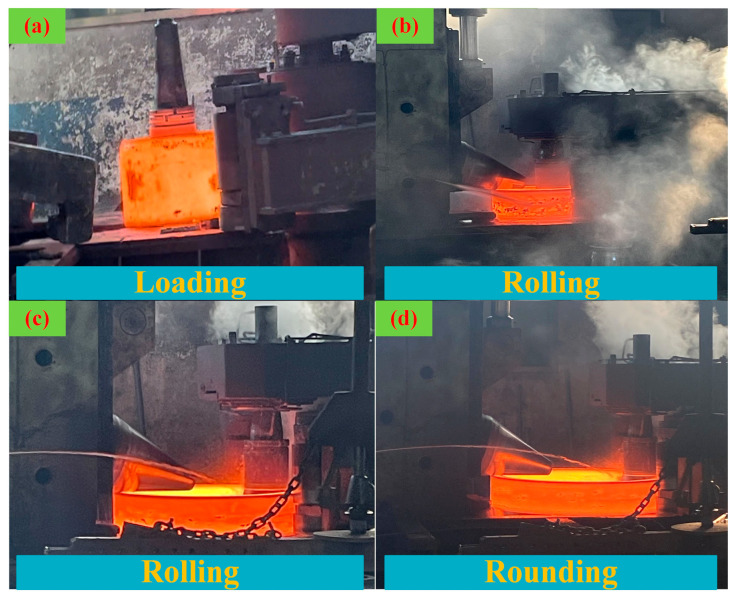
Rolling process of non-standard cross-section bearing rings: (**a**) load the workpiece, (**b**) early stages of the rolling process, (**c**) late stage of the rolling process, (**d**) rounding stage.

To validate the reliability of the simulation, rolling experiments were first conducted on a D53K-4000 CNC radial-axial ring rolling machine at the LYC facility, as shown in Figure 6. The ring rolling blank has the following main geometrical dimensions: an outer diameter of 850 mm, an inner diameter of 500 mm, and a height of 550 mm. The hot bulging speed is 1 mm/s. All other experimental parameters are consistent with the simulation settings described above.

**Figure 7:**
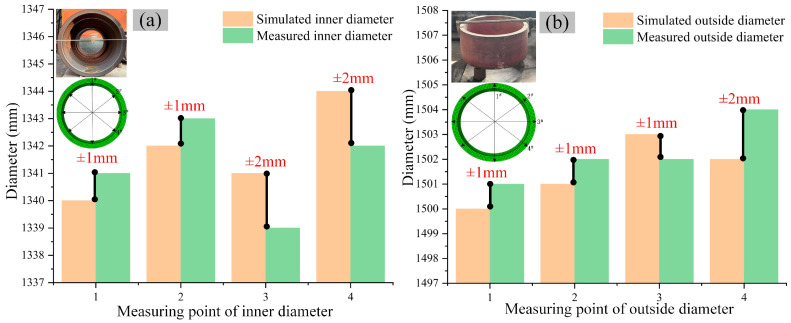
Dimension comparison of experimental and simulated ring results between and after rolling: (**a**) comparison of experimental and simulated inner diameters, (**b**) comparison of experimental and simulated outer diameters.

After the experiment, the inner and outer diameters of the hot-rolled ring were measured at four equally spaced circumferential locations (labeled #1 to #4 with 45° intervals) using a large vernier caliper. Measurements were taken at four uniformly distributed positions on each specimen to mitigate the influence of errors. Within the ABAQUS post-processing module, the built-in distance measurement and query tools are employed. To ensure a direct and equitable comparison with the physical experiment, a virtual measurement protocol is established that fully replicates the experimental methodology. Four equivalent circumferential positions are precisely identified on the simulated rolled ring, after which the software’s measurement tools are used to calculate the distance between diametrically opposite nodes. This process effectively yields the simulated inner and outer diameters at the corresponding cross-sections. The results are shown in Figure 7. The simulation and experimental results indicate that the maximum ellipticity of the outer ring’s inner and outer diameters is 4 mm. The average relative error between the simulated and the experimentally measured values for the key post-rolling dimensions (inner and outer diameters of the outer ring) is less than 1%. This demonstrates that the established rolling simulation model can effectively predict the actual experimental outcomes.

**Figure 8:**
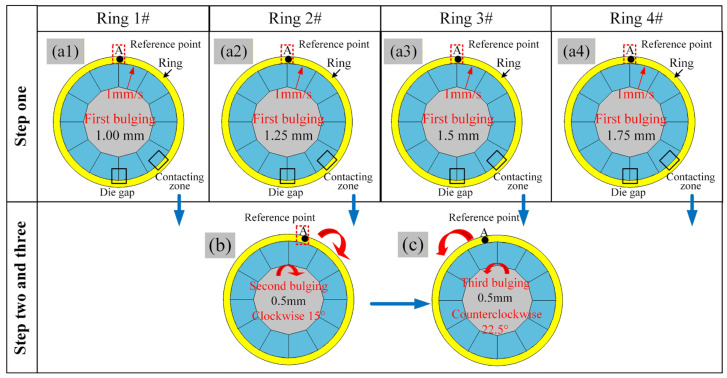
Stepwise flowchart of thermal bulging process for non-circular section bearing rings: (**a1**) first bulging 1 mm, (**a2**) first bulging 1.25 mm, (**a3**) first bulging 1.5 mm, (**a4**) first bulging 1.75 mm, (**b**) second bulging, (**c**) third bulging.

The stepwise bulging process effectively mitigates the accumulation of uneven deformation of the ring by distributing the total deformation across multiple stages, thereby significantly alleviating localized over-thinning and stress concentration. Numerous studies have demonstrated that the bulging amount is the most critical factor influencing the stress–strain state and dimensional accuracy of rings. Consequently, this paper primarily investigates the influence of the bulging amount on the stress–strain behavior and dimensional evolution of rings during forming. The detailed procedure of the stepwise hot bulging process for bearing rings with non-standard cross-sections is illustrated in Figure 7. The process path for the simulation tests is divided into two main stages. In step one, the bulging amounts are set to 1.00 mm, 1.25 mm, 1.50 mm, and 1.75 mm, corresponding to ring sample numbers #1, #2, #3, and #4, respectively, as shown in Figure 8(a1–a4). In step two, the ring is first rotated 15° clockwise, followed by a secondary bulging of 0.50 mm, as illustrated in Figure 8b. Subsequently, it is rotated 22.5° counterclockwise, and a final third bulging of 0.50 mm is completed, as shown in Figure 8c. The four bulging schemes correspond to total bulging amounts of 3.3‰, 3.7‰, 4.1‰, and 4.5‰, respectively.

**Figure 9:**
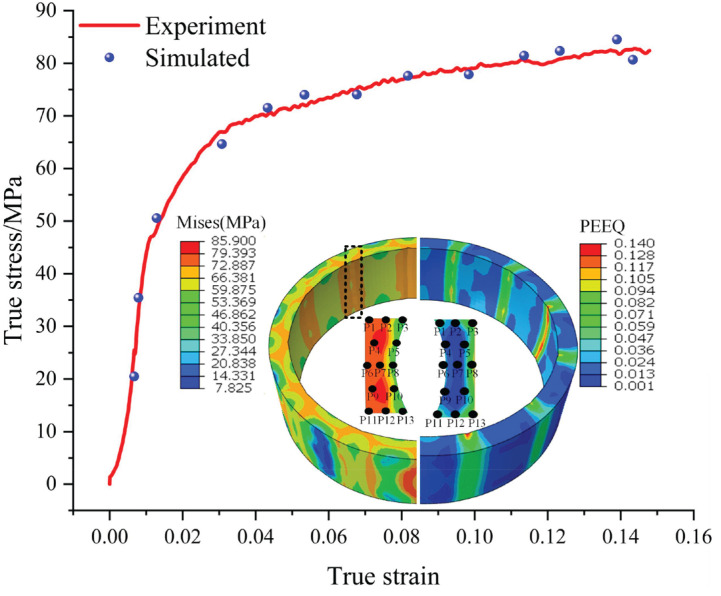
Comparison of simulated and experimented stress and strain of the bearing ring after thermal bulging.

To validate the reliability of the finite element model for the bulging process, 13 representative material points were uniformly selected from the key cross-section of the simulated ring under typical physical experimental deformation conditions (920 °C, 0.01 s^−1^). Thirteen representative material points were selected from the key cross-section of the ring model, covering positions from the inner to the outer regions, to capture the spatial distribution of deformation. To more clearly validate the model accuracy within the actual strain range of the bulging process, the stress–strain states are plotted against the experimental reference curve, which is correspondingly truncated to the maximum bulging strain of 0.16.Their equivalent stress–strain data during the bulging process were extracted, as shown in Figure 9. These data points are plotted and comparatively analyzed in the same coordinate system alongside the material’s true stress–strain reference curve obtained from the hot compression tests. As shown in Figure 9, the simulated stress–strain curves show a high degree of agreement with the experimental curves across the entire deformation range. The experimental value of the peak stress is 82.36 MPa, while the simulated value is 84.55 MPa, yielding a relative error of merely 2.7%. The experimental value of the average stress is 69.60 MPa, while the simulated value is 67.35 MPa, resulting in a relative error of 3.3%. In summary, the high fitness between the simulated data and the experimental reference curves indicates that the developed hot bulging simulation model possesses high predictive accuracy.

**Figure 10:**
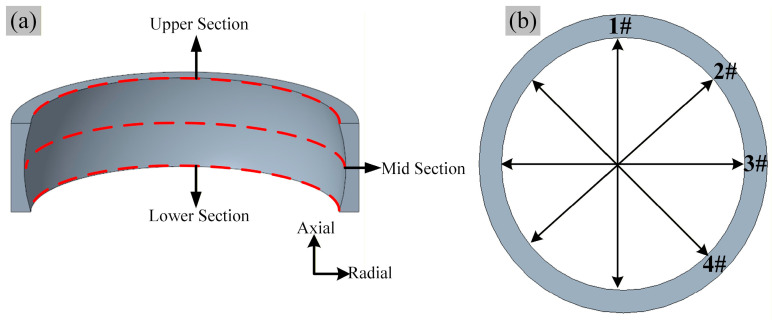
Schematic diagram of diameter measurement for bearing ring after hot bulging: (**a**) select path, (**b**) select points.

To systematically evaluate the effect of different bulging process parameters on the geometric dimensions of the ring, the three representative key cross-sections were selected along the axial direction of the upper, middle, and lower sections, as shown in Figure 10a. To comprehensively reflect the cross-sectional dimensions and reduce random measurement errors, four measurement points were selected on each cross-section for data acquisition, as shown in Figure 10b. The radius at each selected point is calculated using the following formula:

(12) $$r_{i}=\sqrt{{\left(x_{i}-x_{c}\right)}^{2}+{\left(y_{i}-y_{c}\right)}^{2}}$$

The roundness error reflects the deviation of a circular part from its ideal geometric shape. A larger roundness error indicates that the shape of the object deviates more from an ideal circle and vice versa. The roundness error is adopted as the metric for evaluating the dimensional accuracy of the ring after bulging, which can be expressed as

(13) $$Re=r_{max}-r_{min}$$

where *r_max_* represents the maximum diameter of the ring, and *r_min_* represents the minimum diameter e of the ring.

**Figure 11:**
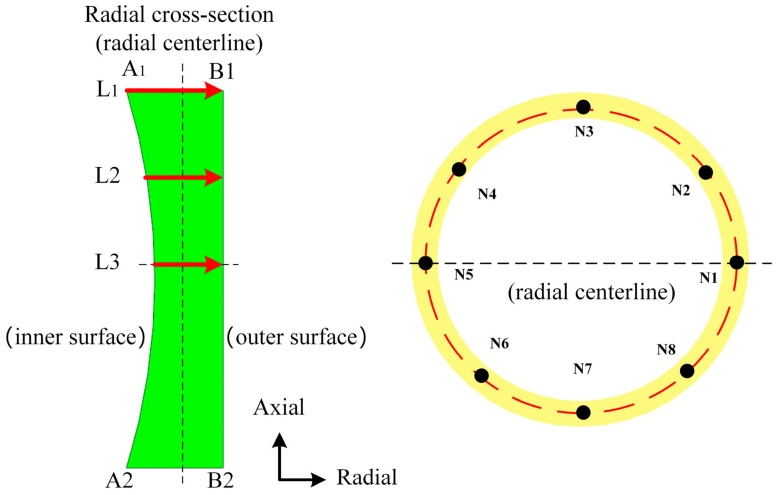
Ring section wall thickness sampling points.

The ring thickness error is recognized as a significant indicator influencing the subsequent machining allowance [34]. As shown in Figure 11, wall thickness measurements are taken at the ring end face, one-quarter of the end face height, and half the end face height. Eight uniformly distributed test positions are designated on each measurement surface with the initial position N1 on the ring set at an angle of 0°. The remaining positions are uniformly spaced at 45° intervals clockwise along the same circumference.

## 4. Results and Discussion

### 4.1. Von Mises Stress Variance of the Bearing Rings During Hot Bulging Process

**Figure 12:**
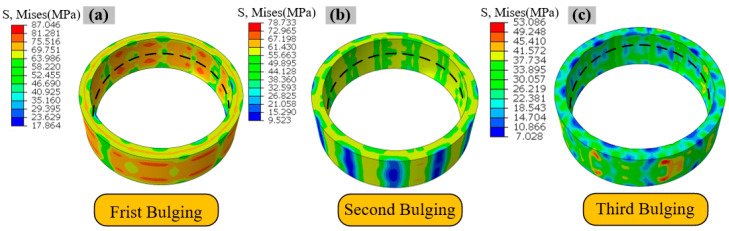
Stress distribution of ring #3 during hot bulging process after unloading: (**a**) first bulging, (**b**) second bulging, (**c**) third bulging.

The periodic “petal-shaped” stress distribution observed after the first bulging step, as shown in Figure 12a [35], fundamentally results from the non-uniform loading mechanism inherent in the bulging process. During initial loading, the regions in direct contact with the bulging head segments experience concentrated plastic deformation, leading to a relatively low stress level with a von Mises stress of 52 MPa. In contrast, the material within the gaps between adjacent segments is strongly constrained by the surrounding deforming material, resulting in significant stress concentration where the von Mises stress rises notably to 84 MPa, thereby forming alternating zones of high and low stress that constitute the petal-like pattern. Figure 12b presents the stress distribution after the second bulging step, which involves a 15° clockwise rotation. At this stage, the originally high-stress gap regions shift to the central contact zones of the bulging heads. Under the new loading conditions, these regions undergo stress relief through plastic deformation, while the previously low-stress contact zones experience renewed constraint and stress evolution. The von Mises stress in these areas decreases from 84 MPa to 45 MPa, representing a reduction of 46%. Figure 12c shows the von Mises stress distribution after the third bulging step, following a 22.5° counterclockwise rotation of the ring. The periodic distribution characteristic further diminishes, and the stress field becomes more uniform. The maximum stress decreases from the previous peak of 78 MPa to 53 MPa, indicating an overall reduction in stress magnitude of 32%. This dynamic, spatially alternating loading process effectively promotes mutual balancing and redistribution between tensile stresses and residual compressive stresses. Consequently, the stress field within the ring progressively homogenizes. The sequential loading and rotation promote stress redistribution through a cyclical process: tensile stresses induced in the current bulging step interact with the pre-existing residual stress field from prior steps. This alternation between tensile loading and subsequent relaxation in rotated positions allows the newly applied tensile stresses and the initial residual compressive stresses to interact and gradually neutralize each other’s concentration effects, thereby promoting a state of mutual equilibrium for the applied tensile stresses and residual compressive stresses [36]. This stepwise bulging strategy effectively promotes the homogenization of von Mises stress distribution.

**Figure 13:**
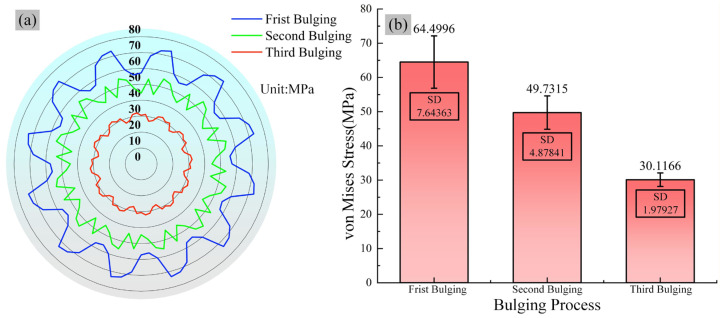
The von Mises Stress distribution and uniformity of bearing ring at different steps across a typical cross-section: (**a**) von Mises stress distribution radar chart, (**b**) von Mises Stress error bar.

As shown in Figure 13a, the circumferential reference path is selected at the central region of the ring to analyze its stress distribution and uniformity. As presented in the radar chart of Figure 13a, the overall stress in the ring exhibits a sequential decrease with an increasing number of bulging steps. The stress ranges for the three bulging stages are approximately 45–65 MPa, 37–55 MPa, and 27–32 MPa, respectively. As the bulging loading continues, the ring material enters a stage of stable plastic flow, during which dynamic recovery and recrystallization occur in its microstructure, leading to a significant weakening of the work-hardening effect. At the same time, the elastic strain energy stored within the material due to prior non-uniform deformation is gradually released, promoting the redistribution of the residual stress field. When the external loading rate reaches a dynamic equilibrium with the internal stress relaxation rate of the material, the overall stress state of the ring stabilizes. Thus, the stress level in the ring gradually decreases and stabilizes. The bar chart with error bars in Figure 13b further quantifies the mean stress and its dispersion after each bulging step. The mean stress is approximately 64.50 MPa after the first bulging step, decreases to 49.73 MPa after the second step, and further reduces to 30.12 MPa after the third step, demonstrating a significant decreasing trend throughout the bulging process. The error bar dispersion indicates a gradual shortening of the error bars as the number of bulging steps increases. The error bars are shortest during the third bulging step, indicating that the stress distribution becomes more uniform, and the forming consistency progressively improves.

**Figure 14:**
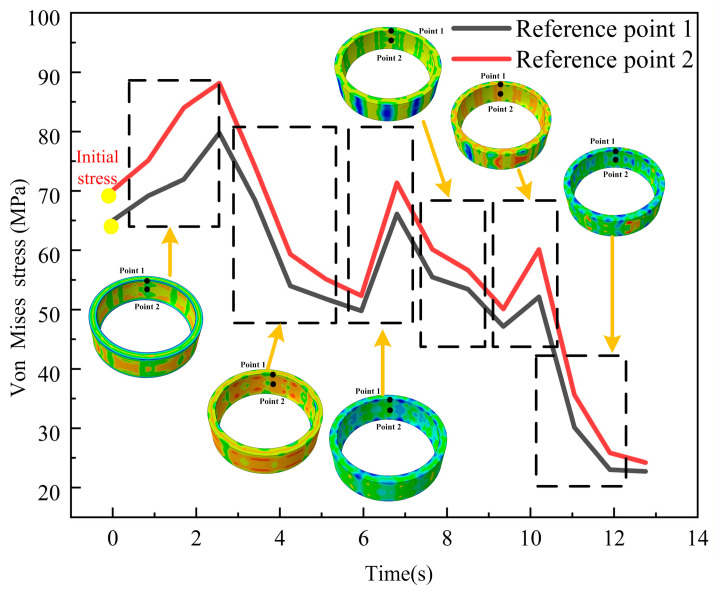
Von Mises stress accumulation history of ring #3 during hot bulging process.

Figure 14 represents the evolution of the equivalent stress distribution for ring #3 during the bulging process. At the onset of each initial bulging stage (2 s, 7 s, and 10 s), a sharp, rapid increase in stress is observed with the maximum stress rising to 89 MPa, 75 MPa, and 65.90 MPa, respectively. This initial surge occurs because the applied load must first overcome the non-uniform residual stress field within the ring, requiring significant stress to bring the material from its heterogeneous initial state to a new yield condition. As the bulging process continues and the applied force stabilizes (6 s, 9 s, and 13 s), the material enters a stage of stable plastic deformation. The work-hardening rate decreases, and the initially unbalanced residual stresses are redistributed and relaxed, evolving toward a stress field that equilibrates with the steady external load. Consequently, the maximum stress decreases to 50 MPa, 48 MPa, and 25 MPa, respectively. Following this, the stress within the ring stabilizes and exhibits a declining trend.

According to the von Mises yield criterion [37], the presence of varying initial stress fields within the ring leads to the formation of a complex and unbalanced residual stress field. This transient stress surge is attributed to a temporary work-hardening effect caused by rapid dislocation multiplication under the instantaneous strain rate, which temporarily exceeds the dynamic recovery rate even at elevated temperatures [38], resulting in a high internal yield strength. Consequently, a substantial circumferential stress must be applied to enable the ring to reach a new yield condition. This results in a sharp increase in the stress level of the ring. In the mid-to-late stages of bulging, plastic flow within the ring stabilizes. The work-hardening rate decreases, and the internal stresses are redistributed and released. A substantial portion of the residual stress is released and redistributed. Once the bulging force stabilizes, the stress of the ring gradually decreases and eventually reaches a steady state.

### 4.2. Strain Variance of the Bearing Ring During Hot Bulging Process

**Figure 15:**
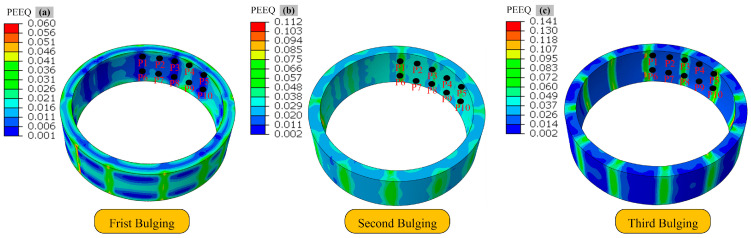
Strain distribution of ring #3 during hot bulging process: (**a**) first bulging, (**b**) second bulging, (**c**) third bulging.

Figure 15 displays the strain distribution of ring #3 after the third bulging step. To quantify the strain uniformity across the cross-section at different stages of hot bulging and understand the strain distribution pattern, 10 tracking points were selected within the rotational range of the ring after its third bulging step in the simulation test group for ring #3.

The effective strain standard deviation of *S_c_* is utilized to characterize the uniformity of effective strain distribution. A smaller *S_c_* value indicates a more uniform distribution of effective strain, and it can be expressed as

(14) $$S_{c}=\sqrt{\frac{1}{N}\sum_{i=1}^{N}{\left(\varepsilon_{i}-\varepsilon \right)}^{2}}$$

where N is the sample number of nodes, *S_c_* is the equivalent strain value at the tracking point, and *ε* is the average equivalent strain of all tracking points.

**Figure 16:**
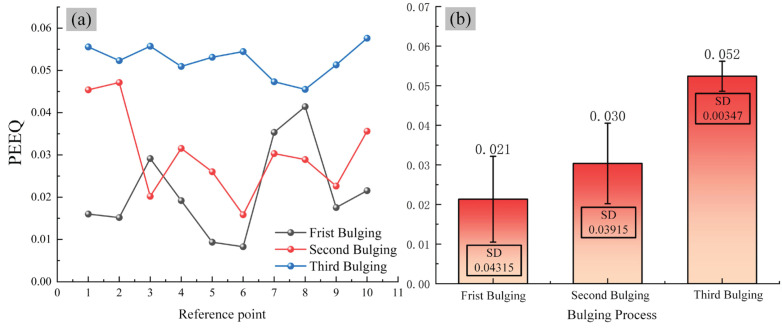
Strain distribution and uniformity characteristics at different steps across a typical cross-section: (**a**) strain distribution, (**b**) strain error bar.

The strain distribution and uniformity characteristics of the reference points across different process stages are shown in Figure 16. A monotonic increase in average strain is observed as the stepwise bulging process advances. The average equivalent strain increases from 0.02131 upon completion of the first step to 0.05239 after the third step, demonstrating a substantial accumulation of plastic deformation in the material. A progressive decrease of 92.0% in strain standard deviation is observed, dropping from 0.04315 to 0.00347. This declining trend demonstrates the effectiveness of the stepwise bulging process in promoting strain homogenization.

**Figure 17:**
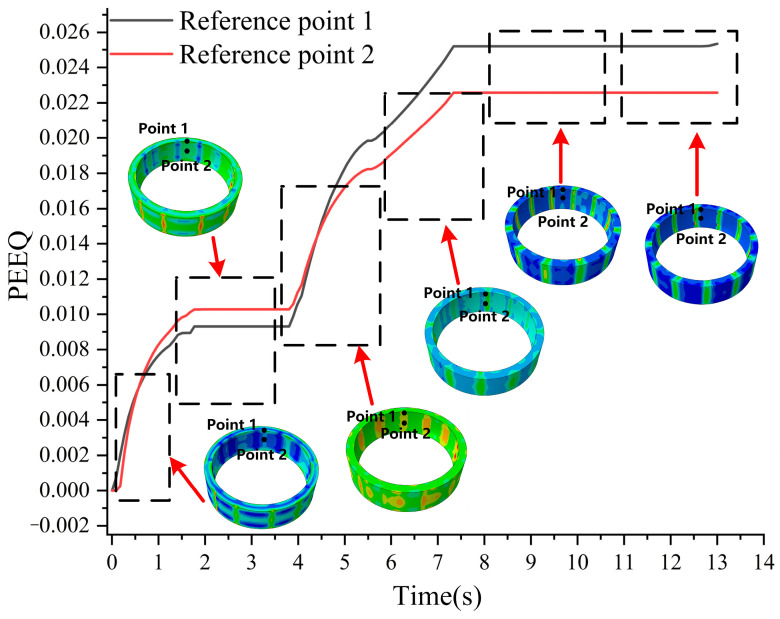
Strain accumulation history of ring #3 during hot bulging process.

As shown in Figure 17, point 1 and point 2 are selected as reference points. These two points exhibit characteristic spatial position transformation. During the initial bulging stage, they are located at different positions within the direct contact zone of the bulging head. Following the subsequent two rotation steps, their relative positions shift to the clearance zone of the bulging head and other locations. Comparing the equivalent strain accumulation history at these two points systematically characterizes the dynamic evolution of strain in the ring during the stepwise rotation bulging process.

Figure 17 presents the cumulative history of strain at the reference points during the three-step rotation bulging process. The strain at the reference points exhibits a monotonic increasing trend throughout the entire process cycle. At the end of the first bulging step, the PEEQ at point 1 accumulates to 0.009. After the second bulging step (with a 15° rotation), it increases to 0.017. Upon completion of the third bulging step (with a −7.5° rotation), it ultimately reaches 0.025. The three bulging contributed strain increments of 0.009, 0.008, and 0.006, respectively, result in a cumulative total of 0.025. The PEEQ at point 2 accumulates to 0.0010 after the first bulging step. Following the second bulging step (with a 15° rotation), it increases to 0.016. After the final third bulging step (with a −7.5° rotation), it reaches 0.022. The three bulging operations contributed strain increments of 0.0001, 0.015, and 0.006, respectively, resulting in a cumulative total of 0.022. Irreversible strain accumulates after each bulging step, confirming the cumulative nature of plastic deformation [39]. The stepwise rotation hot bulging process homogenizes strain distribution, alleviates localized concentration, and achieves a concurrent increase in strain magnitude with improved uniformity.

### 4.3. Deformation Velocity Analysis During Hot Bulging Process

**Figure 18:**
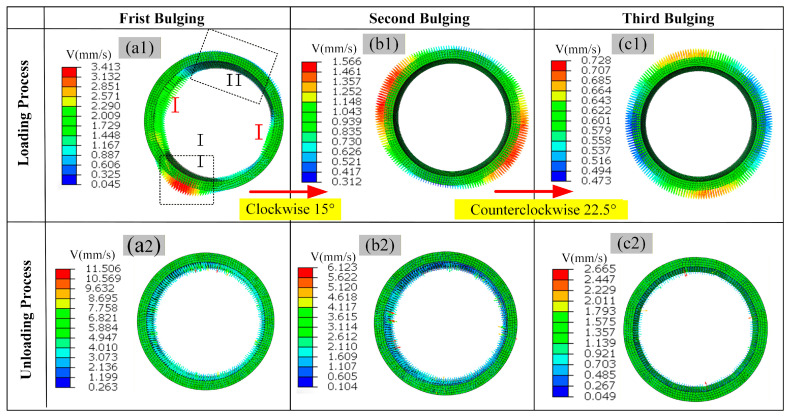
Deformation flow velocity of ring #3 during the bulging process: (**a1**) first bulging, (**a2**) bulging force unloading, (**b1**) second bulging, (**b2**) bulging force unloading, (**c1**) third bulging, (**c2**) bulging force unloading.

Figure 18(a1) illustrates the deformation flow velocity field during the first bulging step. The ring is divided into two regions based on its initial ellipticity: Region I (minor axis of the ellipse) and Region II (major axis of the ellipse). The ring in this region undergoes direct radial compression, resulting in significant plastic deformation, and flows radially outward at a velocity of approximately 3.4 mm/s. In Region II, before the bulging head makes full contact, the ring is primarily subjected to tensile forces transferred from Region I, leading to relatively small radial deformation. It flows radially inward at a velocity of approximately 0.6 mm/s.

Figure 18(a2–c2) present the deformation velocity fields after unloading from the three bulging steps, respectively. Following the first unloading, a reverse flow of material on the inner wall of the ring toward the center occurs, which is circumferentially uniform with an instantaneous velocity of approximately 4.0 mm/s. This velocity decreases to about 2.5 mm/s after the second unloading and it further reduces to about 0.9 mm/s after the third unloading.

Figure 18(b1,c1) show the deformation velocity fields during the second and third bulging steps, respectively. As the number of bulging steps increases, the deformation flow becomes more uniform and stable. During the second bulging, the flow velocity distribution ranges from 0.3 to 1.5 mm/s. By the third bulging, the flow velocity converges to a range of 0.4–0.7 mm/s. The overall flow velocity decreases, and no significant circumferential flow instability is observed in the ring.

During the hot bulging loading stage, the ring undergoes significant elastoplastic deformation. When the external bulging force is removed, the accumulated elastic strain energy is released, driving macroscopic elastic recovery. The inner surface constraint is released first, and unloading rebound primarily occurs on the inner wall of the ring component. The deformation rate of the ring manifests as a uniform centripetal contraction, constituting a radial reverse velocity field. The initial ellipticity and the concentration of internal residual stresses are progressively eliminated through the stepwise rotation hot bulging process. Each application of the bulging force induces synchronized radial plastic flow in the ring. The flow field of the deformation rate is converged, and its stability is confirmed. The geometric accuracy of the ring and its internal stress distribution are optimized simultaneously with the microscopic flow behavior being correlatively linked.

### 4.4. Dimension Evolution of the Bearing Ring During Hot Bulging Process

#### 4.4.1. Roundness Error Analysis

**Figure 19:**
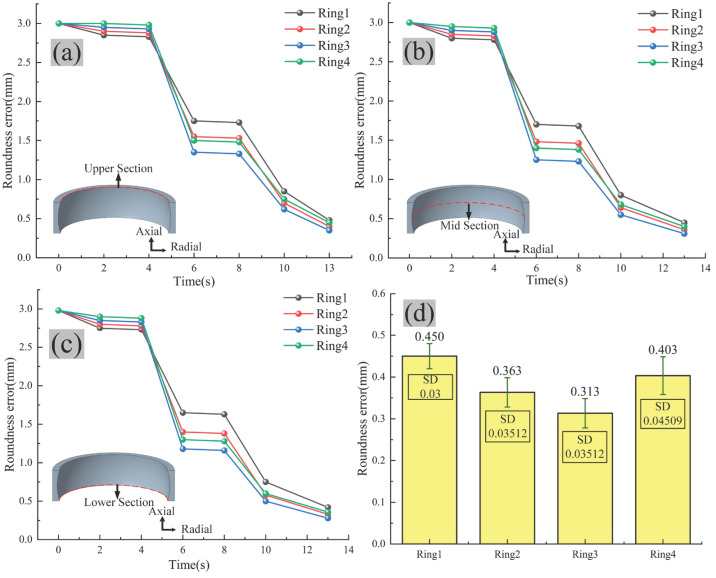
Variation in roundness error at different sections of bearing rings during thermal bulging process: (**a**) upper section, (**b**) middle section, (**c**) bottom section, and (**d**) error comparison.

According to the results presented in Figure 19a–c, when the total bulging amount is 3.3‰, the roundness error after the first bulging step ranges from 2.75 to 2.85 mm, ultimately converging to 0.42–0.48 mm. This trend exhibits the lowest dimensional accuracy among the four tested scenarios. After the total bulging amount is increased to 3.7‰, the roundness error after the first bulging step slightly increases to a range of 2.80–2.90 mm. Following the second bulging step, the roundness error is significantly reduced to 1.38–1.55 mm. The forming precision is improved to 0.33–0.40 mm, which is superior to the previous scheme. When the total bulging amount is 4.1‰, the process performance reaches its optimum. The roundness error after the first bulging step is 2.85–2.95 mm. Following the second bulging step, the roundness is sharply corrected to 1.16–1.35 mm, representing the largest improvement magnitude. The final dimensional accuracy is the highest, with the roundness error consistently stabilized within the range of 0.28–0.35 mm, and the data consistency across different cross-sections is also optimal.

When the total bulging amount is increased to 4.5‰, a decline in performance is observed. The roundness error reaches 2.90–3.00 mm after the first bulging step. Although subsequent steps correct the roundness, the process exhibits increased variability. The final roundness error ranges from 0.36 to 0.45 mm, placing it at a moderate level without further improvement.

Based on the comparison of final roundness errors for the four schemes shown in Figure 19d, the scheme for ring #3 scheme achieves the highest dimensional accuracy. Its average roundness error is 0.313 mm, which is the lowest among the four groups. Ring #3 also exhibits the smallest error bar range, indicating low data dispersion, good repeatability, and the highest process stability. Therefore, considering both geometric accuracy and control stability, the parameters for ring #3 are identified as the optimal process for achieving the highest roundness precision for the ring.

**Figure 20:**
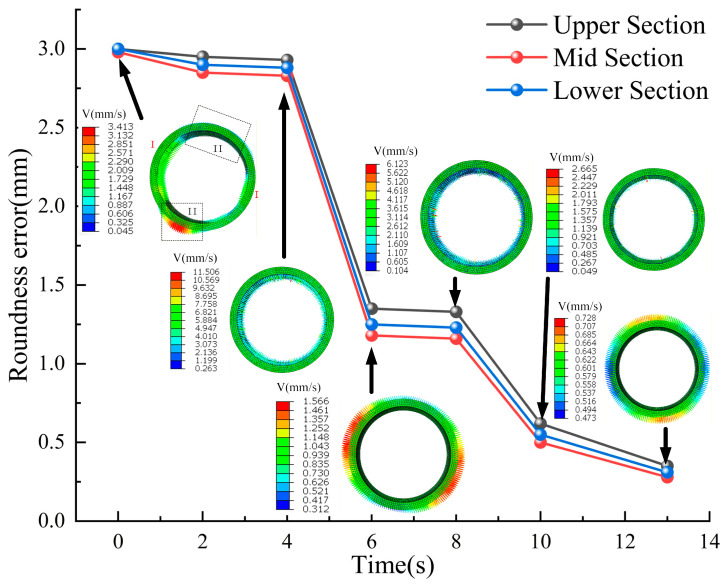
Roundness error accumulation of ring #3 during hot bulging process.

Under the optimal total bulging amount of 4.1‰, Figure 20 illustrates the evolution of roundness error with each step of the bulging process. Due to varying initial bulging amounts across different cross-sections, the initial roundness error of the ring ranges from 2.95 to 3.00 mm. This error corresponds to the significant non-uniformity observed in the deformation flow velocity field. After the initial bulging step, deformation in the minor axis region of the elliptical ring flows outward, while compensatory inward flow occurs in the major axis region. Subsequently, the ring is rotated 15° clockwise and undergoes the second bulging step, resulting in the first significant improvement in roundness error with a reduction of 40–60%. This result indicates that the rotation operation alters the contact phase between the ring and the dies, allowing regions with less deformation in the previous stage to become the dominant deformation zones in subsequent bulging. This enables an active correction of the initial non-uniformity. Finally, after the third bulging step, the roundness error is further converged and stabilized within the target range of 0.28 mm. Through sequential forming, the stepwise rotation hot bulging process effectively achieves a stable transition of the ring from its initial elliptical state to the final high-precision roundness.

#### 4.4.2. Thickness Error Analysis

**Figure 21:**
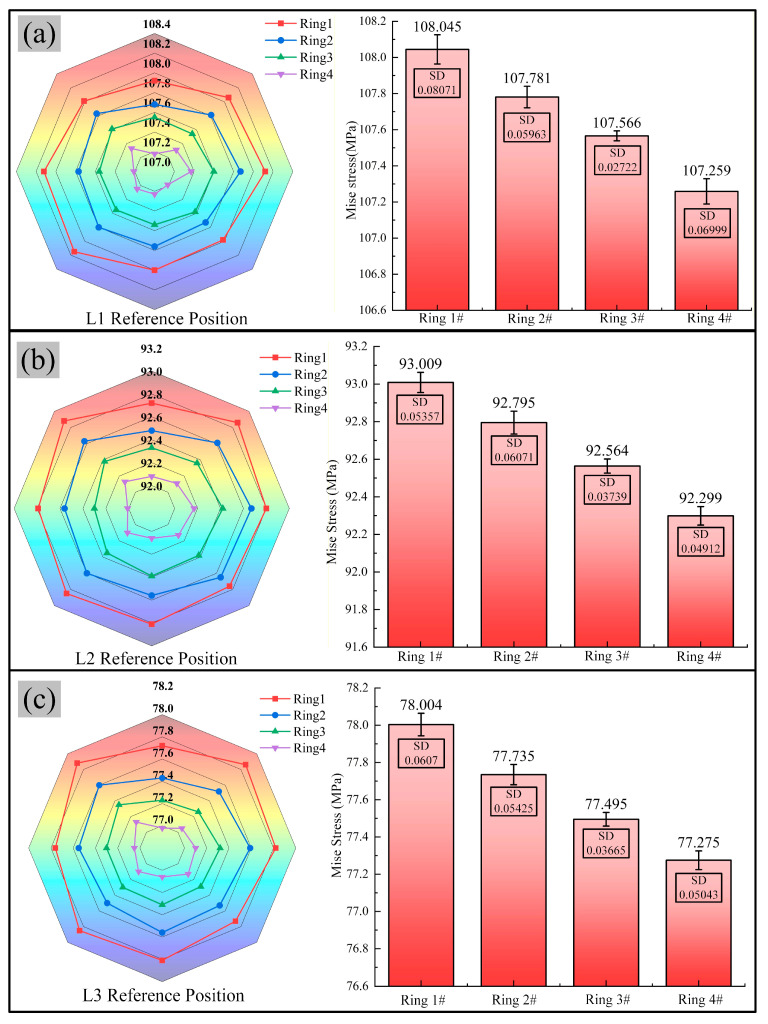
Wall thickness values at sections L1, L2, and L3 on the ring: (**a**) wall thickness at L1 with mean error bars, (**b**) wall thickness at L2 with mean error bars, and (**c**) wall thickness at L3 with mean error bars.

Figure 21 presents the thickness distribution and corresponding uniformity error at locations L1, L2, and L3 (refer to Figure 11) on bearing ring #3. As the first-step bulging amount increases from 1.00 mm to 1.50 mm, the radar chart profile shows a contracting trend, as shown in Figure 20. The overall ring wall thickness systematically decreases, indicating wall thinning during the bulging process. This result aligns with the constant volume principle of plastic deformation. Figure 21 shows that when the first-step bulging amount is 1.50 mm, the error bars are the shortest, indicating the lowest dispersion among the eight measured values for this group. The wall thickness uniformity is superior to that of rings #1, #2, and #4. In contrast, the longer error bars for the 1.00 mm and 1.75 mm groups indicate that both excessive and insufficient deformation lead to uneven wall thickness distribution in the ring and introduce new non-uniformities. This result is consistent with the conclusion from the roundness error analysis that ring #3 achieves the optimal precision, indicating that 1.50 mm represents the key process optimization window in this paper.

Under the 1.50 mm process condition, the strain standard deviation decreases from an initial 0.04315 to a final 0.00347 with a decrease of 92.0%. A uniform strain distribution implies coordinated deformation across different regions of the material, effectively suppressing localized over-thinning. Simultaneously, the maximum equivalent stress in the ring decreases from 70 MPa after rolling to 53 MPa, and the stress distribution becomes more uniform. Residual stress peaks are reduced and homogenized, significantly diminishing the geometric distortion caused by uneven elastic recovery during the unloading stage. A uniform residual stress field promotes consistent rebound across all regions of the ring [40], thereby stabilizing the final dimensions. Therefore, the results indicate that strain homogenization is a necessary condition for optimizing wall thickness distribution, which is achievable by interrupting strain localization paths through the stepwise rotation hot bulging process. Stress homogenization serves as a sufficient condition for dimensional stability, effectively suppressing rebound distortion by reducing residual stress peaks and their gradients.

## 5. Conclusions

This paper investigates the near-net-shape forming process of wind turbine bearing rings with non-standard sections by establishing a finite element model of the continuous hot rolling–bulging process. The stress–strain behavior, flow velocity, and dimension evolution of the ring during the stepwise rotation hot bulging process are analyzed in detail. The main conclusions are summarized as follows:

1. The contact state between the part and the die is periodically altered through the application of the stepwise rotation hot bulging process, enabling stress redistribution and gradual release to homogenize residual stresses. The initial “petal-shaped” high stress of 84 MPa on the rolled ring was significantly relieved by 46%. The maximum equivalent stress of the ring component is decreased by 39% compared to the initial value.
2. The bulging process induces significant accumulated deformation of the bearing ring. The stress concentration is substantially attenuated through a step-by-step loading strategy combined with workpiece rotation. The average equivalent strain of the bearing ring component gradually accumulated from 0.02131 to 0.05239, while its standard deviation decreased significantly by 92.0%.
3. During the initial bulging stage, flow exhibits significant asymmetry due to geometric non-circularity. At the short edge, the outward flow velocity reaches 3.4 mm/s, while at the long edge, the inward flow velocity is 0.6 mm/s. Following the stepwise rotational bulging process, the circumferential flow velocity converged to 0.4–0.7 mm/s, while the unloading process velocity decreased from 4 mm/s to 0.9 mm/s.
4. The process demonstrated optimal efficiency and stability when the initial expansion amount is 1.50 mm, as the secondary and third-step amounts are both 0.50 mm, and the bulging speed is 1.00 mm/s. The roundness error of ring #3 converged steadily from an initial value of approximately 2.9 mm to a range of 0.28–0.35 mm (mean 0.313 mm) after three processing steps. The standard deviation of strain across various sections of ring #3 was reduced by 92.0%, resulting in a significant improvement in wall-thickness uniformity.

### Limitations and Future Work

1. This paper focuses on the hot deformation behavior of GCr15SiMn bearing steel at elevated temperatures; room temperature mechanical properties were not characterized. Future work may supplement these basic mechanical properties (e.g., tensile tests, hardness) according to engineering requirements.
2. The limitations of this paper are as follows: microstructural evolution mechanisms such as dynamic recrystallization, phase transformation, and grain growth were not incorporated into the current FE model, which may affect the accuracy of flow stress and residual stress prediction under high-temperature conditions. This simplification was necessary due to the high computational cost of full-process simulation and the lack of systematically calibrated kinetic parameters for GCr15SiMn. Nevertheless, since all simulation cases share the same constitutive framework, the core conclusions regarding the relative advantages of the stepwise rotation strategy remain robust. Future work will address these limitations through targeted experiments and cross-scale modeling.

## Figures and Tables

**Table 1:** Main chemical composition of GCr15SiMn steel (wt.%).

Elements	C	Cr	Si	Mn	Cu	S	P	Ni	Mo
	0.95~1.05	1.30~1.65	0.40~0.65	0.90~1.25	≤0.25	≤0.020	≤0.027	≤0.003	≤0.001

**Table 2:** Parameter settings for the joint simulation process.

Parameters	Rolling	Bulging
	Values	
Heat transmission coefficient (Ns^−1^mm^−1^ °C^−1^)	10	
Heat radiation coefficient (Ns^−1^mm^−1^ °C^−4^)	0.7	
Heat convection coefficient (Ns^−1^mm^−1^ °C^−1^)	0.02	
Friction coefficient between roll and ring blank μ	0.3	-
Temperature of ring blank T (°C)	1100	-
Temperature of rolls (°C)	50	-
Temperature of environment (°C)	25	-
Rotation of driven roll ω (rad/s) V1	17.5	-
Feed rate of idle roll Vr and axial feed velocity Va (mm/s)	Figure 5b	-
Friction between the bulging slider and mandrel μ_1_	-	0.15
Friction between the bulging slider and ring μ_2_	-	0.5
Return speed of outer slider block (mm/s)	-	0~20
Supporting roll rotational speed (r/min)	-	0~60
Mandrel return speed (mm/s)	-	0~30
Mandrel height (mm)	-	1400
Major edge length of mandrel (mm)	-	259
Minor edge length of mandrel (mm)	-	183

**Table 3:** Mesh convergence analysis results.

Global Mesh Densities	Wall Thickness	Roundness (mm)	Average Stress (MPa)	Strain Uniformity
25	107.56	0.313	30.1166	0.00347
15	107.95	0.308	30.2789	0.00354
5	107.35	0.317	29.9845	0.00342

## Data Availability

The original contributions presented in this study are included in the article. Further inquiries can be directed to the corresponding author.
